# CEEN: A Control Software for Coupled Electrochemistry, EPR, and NMR for Redox Flow Battery and Electrosynthesis Research

**DOI:** 10.1002/mrc.70120

**Published:** 2026-05-22

**Authors:** Vince Elter, Noël de Kler, Giu A. Silva Testa, Zhiyu Zhu, Federico Paruzzo, Simon Bruderer, Evan Wenbo Zhao

**Affiliations:** ^1^ Magnetic Resonance Research Center, Institute for Molecules and Materials Radboud University Nijmegen the Netherlands; ^2^ Bruker Switzerland AG Fällanden Switzerland

## Abstract

We present CEEN (coupled electrochemistry, EPR, and NMR), a Python package designed for coupled and integrated control of Bruker benchtop NMR, benchtop EPR, and Gamry electrochemical workstations. The software enables synchronized acquisition and real‐time visualization of electrochemical, NMR, and EPR data, simplifying operando measurements that traditionally require three independent control interfaces. We demonstrate CEEN through practical applications relevant to aqueous organic and inorganic redox flow battery (RFB) and electrochemical synthesis research. The same setup can be readily used to study any (electrochemical) flow process using magnetic resonance. CEEN streamlines the data acquisition workflow, enabling robust and information‐rich operando NMR and EPR studies.

## Graphical abstract

We present CEEN: a Python‐based control software for coupled electrochemistry, EPR, and NMR for redox flow battery (RFB) and electrosynthesis research. We demonstrate CEEN through practical applications relevant to aqueous organic and inorganic RFB and electrochemical synthesis research. The same setup can be readily used to study any (electrochemical) flow process using magnetic resonance.

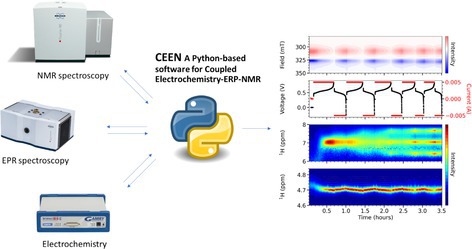

## 1 Introduction

Electrochemistry comprises a wide range of technologies necessary to solve the energy challenges we are facing today [1, 2]. Energy conversion and environmental remediation utilize electrochemical technologies such as batteries, fuel cells, electrosynthesis, and electrocatalysis. In these systems, chemical reactivity is driven by applied potential and current, often giving rise to complex reaction mechanisms, metastable intermediates, and thermodynamics. Gaining molecular‐level insight into such processes under realistic operating conditions is a nontrivial but highly rewarding task.

Redox flow batteries (RFBs) are promising candidates for large‐scale stationary energy storage due to their decoupled power and energy densities, flexible design, and long cycle life [3]. However, the development of sustainable, cost‐effective RFB electrolytes remains challenging. Degradation reactions, pH drift, and the formation of transient intermediates often occur under realistic operating conditions and can strongly impact capacity retention and efficiency [4, 5, 6, 7, 8, 9, 10, 11]. Many of these processes are difficult to study using conventional ex situ analytical techniques, which in many cases lack either chemical specificity or temporal resolution [4].

Magnetic resonance techniques—including nuclear magnetic resonance (NMR) and electron paramagnetic resonance (EPR)—are noninvasive, atom‐specific, and quantitative, and can be applied to both liquid‐ and solid‐state materials. These characteristics make magnetic resonance ideally suited for operando studies of electrochemical devices and for establishing structure–function relationships under realistic operating conditions [12].

Operando magnetic resonance is particularly useful for detecting metastable intermediates, monitoring reaction kinetics, and establishing trigger–response relationships during evolving thermodynamic processes [13, 14, 15, 16]. A possible mode to perform operando measurements with NMR and EPR is in flow mode, consisting of flowing the electrolytes through detection regions in dedicated NMR and EPR flow cells. This setup is information‐rich as it leverages the complementarity of NMR and EPR. EPR provides insight into the chemical identity, electronic structure, and concentration of paramagnetic species such as organic radicals and certain transition‐metal complexes, whereas NMR enables identification and quantification of nuclei such as ^1^H, ^7^Li, and many others across the periodic table in diamagnetic species. When combined, EPR and NMR yield a comprehensive picture of the redox chemistry taking place in the electrolytes of RFBs, and have proven useful for both organic and inorganic redox‐active materials [17]. Benchtop NMR and EPR spectrometers offer a cost‐effective alternative to high‐field instruments while retaining sufficient sensitivity for real‐time studies. Benchtop operando NMR and EPR methodologies have been demonstrated for elucidating redox mechanisms and ion transport [17, 18, 19].

Besides RFBs, coupled magnetic resonance–electrochemical measurements have increasingly been explored in electrocatalysis. NMR has been applied to monitor electrolyte composition and reaction intermediates under realistic electrochemical conditions, for example, high‐current electrochemical CO_2_ reduction studies using inline benchtop flow cells and lithium‐mediated ammonia synthesis using an in situ cell [20, 21]. Operando EPR combined with electrochemical control has also enabled real‐time tracking of radical intermediates in catalytic systems [22].

Despite the great analytical potential, coupled operando electrochemistry–EPR–NMR experiments present practical challenges. Each instrument—NMR spectrometer, EPR spectrometer, and potentiostat that controls the electrochemistry—is operated through its own software. As a result, experiments must be initiated separately, parameters are stored independently, and data are inherently asynchronous. This fragmented workflow complicates data synchronization, increases the burden on the researcher, and can limit accessibility and reproducibility unless experimental conditions are documented meticulously. With benchtop instruments becoming more advanced and modular, there is a growing demand for streamlined methods enabling automatically coupled electrochemistry–EPR–NMR.

Here, we introduce CEEN—coupled electrochemistry, EPR, and NMR—a Python package that unifies control of a Gamry potentiostat and Bruker benchtop NMR and EPR spectrometers. Our goal is to create an intuitive research tool for nonspecialists in NMR and EPR. CEEN enables synchronized data acquisition across all three platforms, eliminating the need for manual alignment or interpolation during post‐processing. The software also facilitates complex acquisition schemes, such as alternating pulse sequences to generate multiple pseudo‐2D NMR datasets in parallel, or constructing pseudo‐3D datasets using multidimensional sequences with extractable 1D spectra. In this paper, we describe the architecture and functionality of CEEN and demonstrate its application in monitoring redox processes in aqueous (metal)organic RFB systems and electrosynthesis.

## 2 Software

### 2.1 General Information

The CEEN Python package, along with documentation and example scripts, is available through the project's public repository (https://github.com/NMReChem‐Group/CEEN). Information regarding installation, code dependencies, and experimental setup can all be found on the GitHub page. In this paper, we only give an overview of what CEEN is like, and what it can be used for.

The current implementation has been developed and tested for the following instruments: Bruker Fourier80 benchtop NMR spectrometer (80 MHz), Bruker ESR5000 benchtop EPR spectrometer (X‐band, 9.45 GHz), Gamry Interface1010E potentiostat. Other Gamry potentiostats are expected to function without any modifications, but this has not been verified.

For NMR, it is necessary to have a Bruker spectrometer using TopSpin, including the Bruker TopSpin Python API (available starting from TopSpin 4.3). For EPR, it is necessary to have an ESR5000 including installation of the ESRStudio software and the ESRStudio Software Development Kit (SDK). Please note that all instances of ESRStudio, including any background processes, must be closed before running the Python script; this can be done using the Windows Task Manager and terminating any processes related to ESRStudio.

CEEN is currently under development and is intended as a flexible research framework rather than a finalized software product. Although the present implementation provides reliable synchronized control of NMR, EPR, and electrochemical instrumentation for the use cases demonstrated here and more, additional functionality and extended instrument support remain areas for future development. As CEEN is applied to a broader range of experiments, limitations and opportunities for expansion are expected to become apparent, guiding ongoing refinement of the software.

### 2.2 Setting up an Experiment

Before initiating an experiment, each instrument must be configured appropriately. For the NMR spectrometer, the TopSpin Network Interface must be running. The EPR spectrometer requires that ESRStudio is fully closed, including any background processes. For the potentiostat, the cell should be off (indicated by a light on the housing), which can be ensured by performing a simple restart. There is a script to help diagnose issues with the APIs if they are encountered.

CEEN manages data acquisition using a JSON configuration file—hereafter referred to as the setup file. This file contains all relevant parameters for NMR, EPR, and potentiostat control, as well as global experimental settings such as maximum experiment duration, synchronization options, and device toggles. The setup file also serves as a record of the experimental parameters, facilitating reproducibility and post‐experiment review.

For magnetic resonance measurements, CEEN generates a series of 1D spectra based on the parameters stored in the setup file. The NMR module can alternate between multiple pulse sequences to acquire several pseudo‐2D experiments in parallel. Although higher data density could be achieved using dual‐nucleus detection, this functionality is not currently implemented. Sequence alternation offers one advantage over dual‐nucleus detection: it allows different pulse sequences targeting the same nucleus as well as more complex pulse sequences, which will be briefly discussed later in this article.

**FIGURE 1:**
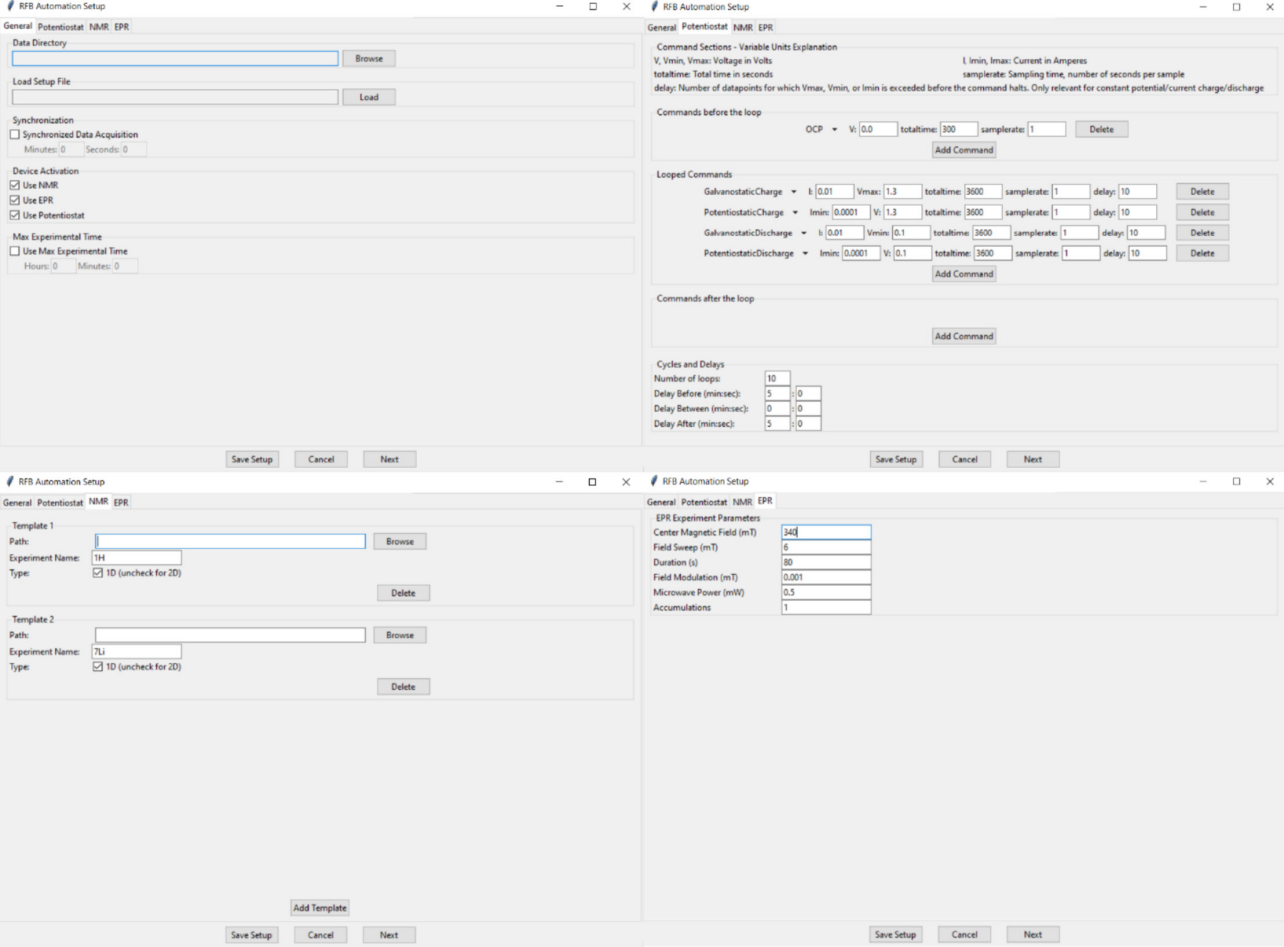
GUI of the CEEN software. Each instrument has a dedicated configuration tab to define the parameters of the corresponding measurement.

Data acquisition can be initiated either by manually writing the setup file or via the graphical user interface (GUI). In both cases, a setup file is generated. The GUI (Figure 1) is minimalistic but helps prevent syntax errors that may arise when editing JSON files manually. Even a minor formatting mistake—such as a misplaced comma or bracket—can render the setup file unreadable. In more critical scenarios, a typographical error might only be caught after the potentiostat has already altered the electrolyte composition, potentially compromising the entire experiment irreversibly. Although such cases are rare, the GUI eliminates this risk.

#### 2.2.1 Acquisition

After the experimental setup is built, and all devices and APIs are configured properly as detailed on the GitHub page: https://github.com/NMReChem‐Group/CEEN, an experiment can be set up. The setup_experiment.bat file can be launched to start an experiment. It opens a GUI that guides the user through the set‐up process. As shown in Figure 1, the “Data Directory” field defines the location where the setup file and all acquired data will be saved. The “Load Setup File” field allows the user to import an existing setup file or template; its contents are displayed in the GUI, enabling inspection, modification, or exact replication of a previous experiment. The general settings tab contains the following parameters:

##### 2.2.1.1 Synchronization

**FIGURE 2:**
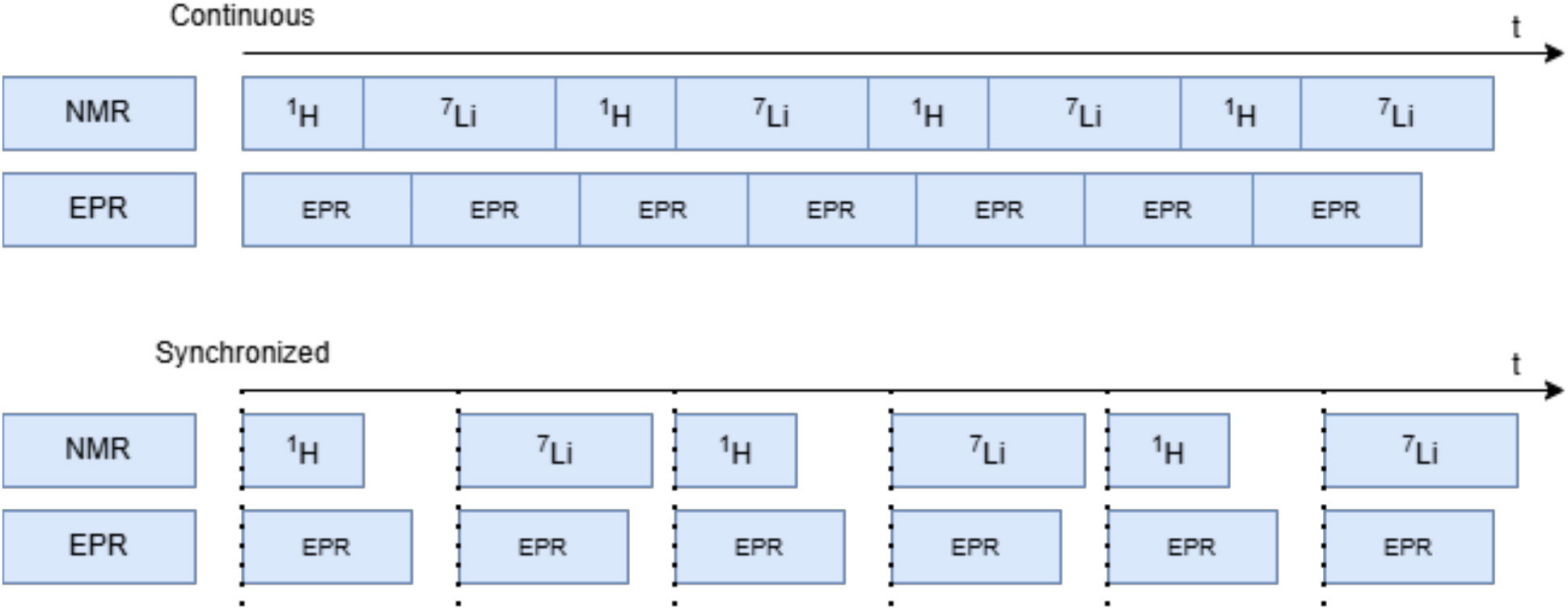
Schematic representation of synchronization. Electrochemistry is not pictured as it is unaffected. The blocks' content and width correspond to their measurement type and duration, respectively. Measurement times are arbitrary and serve illustrative purposes only.

Allows the user to specify a time interval which defines the acquisition interval for NMR and EPR measurements. A schematic representation is found in Figure 2. When synchronization is active, both spectrometers remain idle until the defined interval has elapsed since the previous measurement request. Experimental parameters for each instrument may be adjusted to match the desired acquisition time. For example, increasing the number of accumulations of the faster measurement can ensure that its acquisition time more closely matches that of the slower measurement, thereby minimizing idle time.

##### 2.2.1.2 Device Activation

Allows the user to select which instruments will be used during the experiment. These selections also determine which configuration tabs are displayed.

##### 2.2.1.3 Max Experimental Time

Defines a firm upper limit for the potentiostat operation. If the maximum duration is exceeded, the potentiostat receives a command to stop immediately and save all collected data. Only after this process is completed do the NMR and EPR modules receive the signal to halt acquisition.

Each instrument has a dedicated configuration tab as shown in Figure 1. The potentiostat settings are conceptually similar to those available in Gamry Framework but are streamlined for battery‐relevant cycling protocols. Users may define arbitrary cycling sequences, protocols before and after, and timing delays. The EPR configuration mirrors the 1D acquisition parameters available in ESRStudio. The NMR configuration differs markedly from TopSpin: rather than defining acquisition parameters directly in the GUI, the user specifies a path to a previously executed 1D experiment. CEEN reads the acquisition parameters from this reference dataset and produces a pseudo‐2D spectrum by assembling a series of 1D acquisitions recorded at uniform time intervals. It is recommended to perform a single NMR reference measurement with the selected pulse sequence immediately prior to starting the experiment. Incorrect referencing—such as an offset introduced by the previous user—may otherwise shift the spectral window, limiting the effectiveness of the real‐time plotting interface.

Alternated Multiple Detection (AMD) is a method implemented in CEEN to enable simultaneous acquisition of multiple 2D NMR datasets. During the experiment, the software cycles through the given pulse sequences, recording (or extracting for more complex pulse sequences) a single 1D spectrum from each pulse sequence. These 1D spectra are then grouped according to the name given to them, so it is important to give them unique names. After the experiment, they are assembled into full pseudo‐2D spectra, albeit with reduced sampling density for each pulse sequence.

Pressing “Save” generates a new setup.json file in the selected Data Directory, overwriting any file with that name that already exists without warning. From here, the user can easily retrieve all experimental parameters, if for instance the experiment needs to be restarted. Pressing “Next” executes the same “Save” logic, then closes the configuration interface and opens the real‐time plotting settings window. In this window, the researcher may select which signals to display: for the potentiostat, current and voltage; for NMR and EPR, any number of user‐defined spectral regions. Pressing “Start Experiment” initiates the experiment and opens an initially empty plot window that populates as data become available. To preserve memory and maintain readability during long experiments, the live plots display only the most recent 6 h of acquired data.

For both NMR and EPR measurements, the GUI displays contour plots representing the most recent 6 h of data. In addition, line plots of the current and voltage recorded by the potentiostat are presented for the same time window. The GUI also includes reminders to verify proper hardware configuration and a button to safely terminate the experiment.

#### 2.2.2 Processing

After data acquisition is completed, for NMR spectra, data processing can be performed using either Topspin or ssNake [23]. To use ssNake, open and combine all the spectra in Bruker format, and process. The user can then export the finalized 2D NMR spectrum as a .csv file. It should be named identically to the corresponding pulse sequence. If a corresponding .npz file already exists, it must be deleted to ensure regeneration using the ssNake‐processed data. We have found no need to process EPR spectra.

Then the generate_figures.bat file can be executed to launch the processing GUI, which facilitates the generation of figures from the acquired datasets. The user must first select a setup file associated with a completed experiment; CEEN then identifies all available NMR, EPR, and electrochemical datasets. The acquired 1D spectra are assembled into 2D datasets and stored as .npz files for efficient downstream access. For each data type, the user may select which signals or spectral regions to plot and how they should be displayed, using options analogous to those in the real‐time plotting interface.

## 3 Use Cases

Three case studies on RFB and electrosynthesis using CEEN are presented here: 1) an aqueous Cu/Fe RFB; 2) an anthraquinone/ferrocyanide RFB; 3) TEMPO‐mediated alcohol oxidation. Because this article aims to showcase the usage of CEEN, in‐depth discussions of the chemistries associated with each system will be published elsewhere.

### 3.1 Case 1: Aqueous Inorganic RFB

**FIGURE 3:**
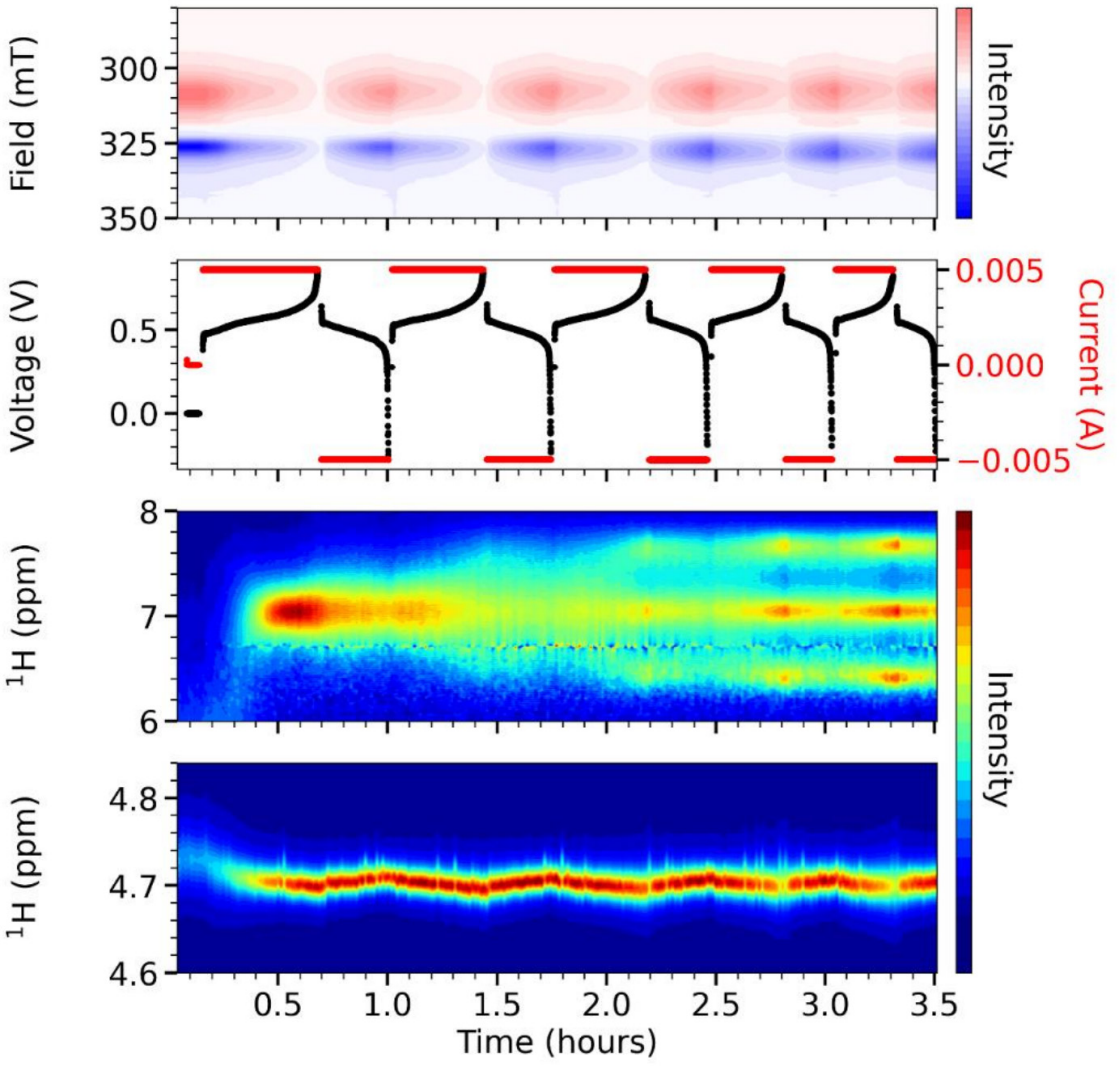
Operando ^1^H NMR and EPR spectra of a Cu/Fe RFB. From top to bottom: (1) Contour plot of EPR spectra corresponding to Cu(II) complexes in solution. Pink = strong; blue = weak intensity. (2) Charge and discharge curves of the RFB with constant current (5 mA, 1 mA · cm^−2^), cut‐off voltage (0.8 V for charging and 0.1 V for discharging, 10 points below). (3) Contour plot of ammonium NMR signals. (4) Contour plot of H_2_O NMR signal. Red = strong; blue = weak intensity.

In this case study, it was demonstrated that operando EPR and NMR can be used to study Cu/Fe RFBs. In most studies, only the negolyte or posolyte is studied by operando methods; however, this provides limited information about the RFB performance and chemistry [4]. That is why, in this case study, operando EPR and NMR were decoupled to monitor the chemistry of the negolyte (Cu) and posolyte (Fe) in a single experiment. As the negolyte, the [Cu(2,2′‐bipyridine)_2–3_]^2+^ complex was used and sulfonated ferrocene (FcSO_3_) as the posolyte [24, 25]. The RFB was operated at a constant current of 5 mA (1 mA · cm^−2^) with a 0.8‐V upper voltage limit. The results are shown in Figure 3.

The Cu(II)/Cu(I) redox pair in negolyte solution was monitored by EPR spectroscopy, in which the Cu(II) concentration can be quantified to measure the state of charge (SoC). During charging, the EPR signal intensity decreased, consistent with the electrochemical reduction of paramagnetic Cu(II) to diamagnetic Cu(I). During discharge, the signal increased again, corresponding to the reformation of Cu(II). These reversible changes demonstrate that the SoC can be tracked using EPR.

In the same experiment, the Fe(II)/Fe(III) redox couple was monitored by operando NMR to qualitatively assess the stability of the FcSO_3_ complexes. Over several charge–discharge cycles, the NMR spectra exhibited irreversible spectral features, most notably in the resonances of ammonium cations, which are present as the supporting electrolyte. The chemical shift and peak shape of the ammonium signal are strongly pH‐dependent and can only be observed in acidic conditions. After the first cycle, the ammonium resonance appeared as a single peak. After the second cycle, the ammonium signal changed to a triplet due to a significant decrease of the pH value to approximately 3, reflecting a transition from the fast‐exchange to the slow‐exchange regime. From these results, it was concluded that the capacity fade of the battery is mainly caused by side reactions that occur in the posolyte solution.

### 3.2 Case 2: Aqueous Organic RFB

The role of supporting electrolytes in RFBs extends beyond simply providing ionic conductivity; as charge‐balancing ions can actively influence electrochemical performance, solubility, and reaction equilibria. In previous work, we monitored the behavior of charge‐balancing Li^+^ in solution to elucidate its transport behavior in a symmetric flow cell [19]. Following this work, we applied a similar methodology to a pH‐neutral aqueous organic RFB employing anthraquinone‐2,7‐disulfonic acid (2,7‐AQDS) as the anolyte, paired with a mixture of lithium ferricyanide/ferrocyanide (Li_3_Fe(CN)_6_/Li_4_Fe(CN)_6_) as the catholyte. A solution of LiCl was chosen as the supporting electrolyte. In this case study, the goal was to investigate how Li^+^ ions behave during battery cycling and how their interactions with redox‐active species evolve under operating conditions.

**FIGURE 4:**
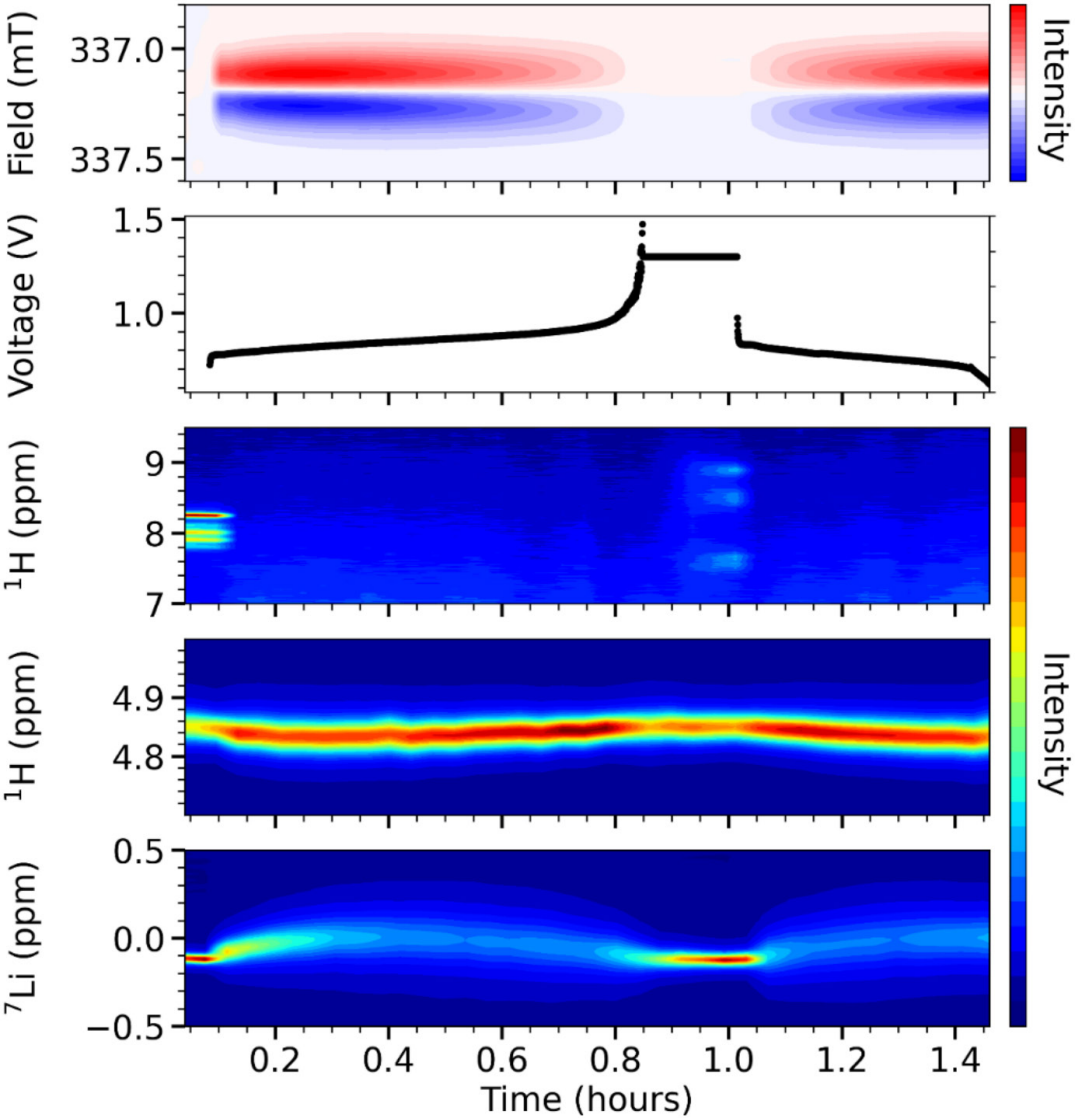
Operando ^1^H and ^7^Li NMR and EPR spectra of a 2,7‐AQDS/Fe RFB. From top to bottom: (1) Contour plot of EPR spectra corresponding to the radical intermediate of 2,7‐AQDS. Pink = strong; blue = weak intensity. (2) Charge and discharge voltage curves of the RFB with constant current (50 mA, 10 mA · cm^−2^), voltage limit (1.3 V), voltage hold (1.3 V), and discharge (0.6 V, 5 points below). Contour plots of (3) 2,7‐AQDS, (4) H_2_O, (5) ^7^Li NMR. Red = strong; blue = weak intensity.

AMD was performed to acquire both ^1^H and ^7^Li pseudo‐2D NMR spectra. This dual NMR acquisition enables simultaneous monitoring of both the supporting electrolyte and the redox‐active organic species, providing complementary mechanistic insight. The results are shown in Figure 4. The reduction of 2,7‐AQDS proceeds through two sequential one‐electron steps and involves a radical intermediate, which is also generated through comproportionation between the oxidized and the fully reduced forms [26]. Because these intermediates are paramagnetic, they influence both EPR and NMR spectra, appearing as characteristic EPR signals and inducing line broadening and chemical‐shift perturbations in NMR.

During charging, the EPR signal increased as the radical concentration rose, while the NMR spectra showed progressive broadening and shifting of key resonances. These effects intensified until the battery reached 50% SoC, after which they diminished as the system approached the fully reduced state. At 100% SoC, both the EPR signal and the NMR line broadening and chemical‐shift perturbations disappeared, consistent with the full reduction of the radical species.

The ^7^Li NMR spectra specifically probe the Li^+^ ions and allow tracking of their local chemical environment. Variations in chemical shift and linewidth provide evidence of dynamic interactions between the Li^+^ ions and the generated radical intermediates. A more detailed analysis of these interactions is beyond the scope of the present manuscript and will be addressed in a future study. The shifts observed in the ^1^H NMR resonances are consistent with previously reported behavior for anthraquinone‐based redox systems and reflect changes in the electronic structure of the molecule upon reduction [9, 14, 17, 27]. Accordingly, these new signals appearing at approximately 0.9 h correspond to the fully reduced form of 2,7‐AQDS.

### 3.3 Case 3: Electrochemical Oxidation of 2‐Phenoxy‐1‐Phenylethanol

**FIGURE 5:**
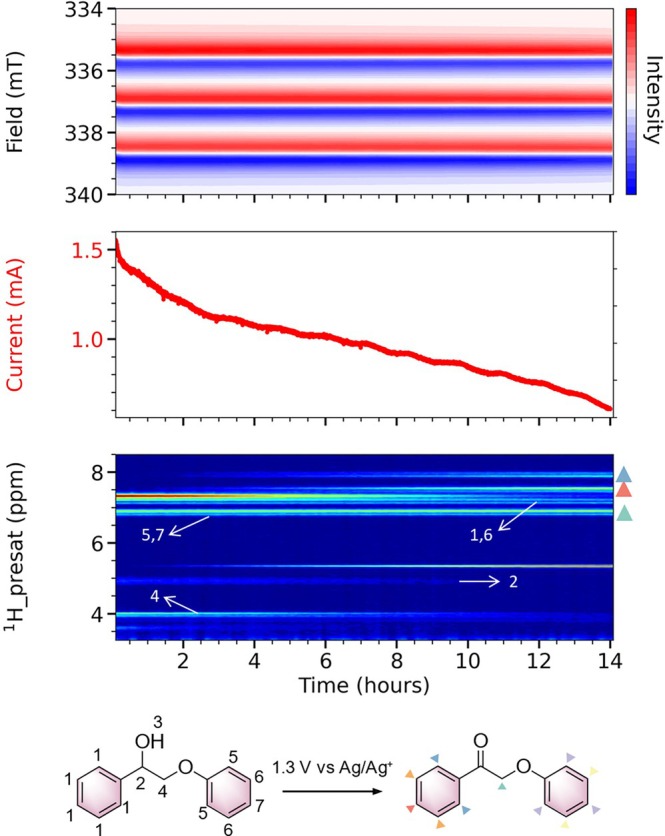
Electrochemical oxidation of 2‐phenoxy‐1‐phenylethanol (pp‐OH) in acetonitrile. From top to bottom: (1) Contour plot of EPR spectra corresponding to TEMPO. Pink = strong; blue = weak intensity. (2) Current profile of the electrolyzer at 1.3 V vs. Ag/Ag^+^. (3) Contour plot of pp‐OH and phenyl benzoate ^1^H NMR resonances and the corresponding signal assignment. Red = strong; blue = weak intensity.

In this case study, bulk electrolysis was performed on 2‐phenoxy‐1‐phenylethanol (pp‐OH) to convert it to phenyl benzoate via oxidation of the benzylic hydroxyl group. NMR can provide molecular fingerprints of pp‐OH and its reaction products, but cannot capture transient intermediates or the dynamic behavior of catalytic mediators such as TEMPO in real time. Combining NMR with EPR allows operando monitoring of the redox‐active TEMPO mediator, which plays an essential role in lignin oxidation, alongside the evolving substrate and product concentrations. The results are shown in Figure 5.

The solution consisted of 5 mM TEMPO, 10 mM pp‐OH, and 100 mM tetrabutylammonium hexafluorophosphate (TBAPF_6_) in acetonitrile. TEMPO served as the redox mediator and TBAPF_6_ as the supporting electrolyte. Chronopotentiometry was performed at 1.3 V vs. Ag/Ag^+^ for 14 h. A pre‐saturation (Pre‐SAT) pulse was applied to suppress the solvent signal to improve detectability of low concentration pp‐OH and phenyl benzoate [28].

Throughout the experiment, the proton resonances of pp‐OH gradually decreased in intensity, while the corresponding resonances of the product, phenyl benzoate, increased. The EPR signal of TEMPO exhibited only minor changes, with slight line narrowing during the course of electrolysis. The current decreased from approximately 1.5 mA (0.3 mA cm^−2^) at the start of the experiment to 0.6 mA (0.12 mA · cm^−2^) by the end.

## 4 Conclusion

We have introduced CEEN, a Python interface developed to control multiple analytical instruments, including benchtop NMR, benchtop EPR, and potentiostat. This approach streamlines operando experimentation, improves synchronization of data, and simplifies data handling, post‐processing, and real‐time visualization. CEEN provides a practical and flexible platform for coupled electrochemical–spectroscopic studies in RFBs and electrosynthesis. Beyond the examples presented here, CEEN is readily applicable to other electrochemical and spectroscopic systems.

## Author Contributions

**Vince Elter:** software, methodology, validation, visualization, writing – original draft, writing – review and editing. **Noël de Kler:** validation, investigation, methodology, writing – original draft, writing – review and editing. **Giu A. Silva Testa:** methodology, validation, investigation, writing – original draft, writing – review and editing. **Zhiyu Zhu:** methodology, investigation, validation, writing – original draft, writing – review and editing. **Federico Paruzzo:** methodology, software, supervision, writing – original draft, writing – review and editing. **Simon Bruderer:** methodology, software, supervision, writing – review and editing, writing – original draft. **Evan Wenbo Zhao:** conceptualization, supervision, funding acquisition, project administration, resources, writing – original draft, writing – review and editing.

## Funding

This work was supported by Nederlandse Organisatie voor Wetenschappelijk Onderzoek OCENW.M.21.308, IMP.EXP.23‐24.059, NWA.1418.24.005, 184.035.002.

## Data Availability

The data that support the findings of this study are available from the corresponding author upon reasonable request.
